# Effect of Calcium Precursor on the Bioactivity and Biocompatibility of Sol-Gel-Derived Glasses

**DOI:** 10.3390/jfb10010013

**Published:** 2019-02-23

**Authors:** Alejandra Ruiz-Clavijo, Andrew P. Hurt, Arun K. Kotha, Nichola J. Coleman

**Affiliations:** 1 Facultad de Ciencias Químicas, Universidad Complutense de Madrid, Av. Complutense, 28040 Madrid, Spain; aruizdeclavijo@gmail.com; 2 Faculty of Engineering and Science, University of Greenwich, Chatham Maritime, Kent ME4 4TB, UK; a.p.hurt@gre.ac.uk (A.P.H.); arunkotha@yahoo.com (A.K.K.)

**Keywords:** bioactive glass, calcium silicate, guided tissue regeneration, periodontal repair, Stöber process, chitosan, MG63 osteosarcoma

## Abstract

This study investigated the impact of different calcium reagents on the morphology, composition, bioactivity and biocompatibility of two-component (CaO-SiO_2_) glasses produced by the Stöber process with respect to their potential application in guided tissue regeneration (GTR) membranes for periodontal repair. The properties of the binary glasses were compared with those of pure silica Stöber particles. The direct addition of calcium chloride (CC), calcium nitrate (CN), calcium methoxide (CM) or calcium ethoxide (CE) at 5 mol % with respect to tetraethyl orthosilicate in the reagent mixture gave rise to textured, micron-sized aggregates rather than monodispersed ~500 nm spheres obtained from the pure silica Stöber synthesis. The broadening of the Si-O-Si band at ~1100 cm^−1^ in the infrared spectra of the calcium-doped glasses indicated that the silicate network was depolymerised by the incorporation of Ca^2+^ ions and energy dispersive X-ray analysis revealed that, in all cases, the Ca:Si ratios were significantly lower than the nominal value of 0.05. The distribution of Ca^2+^ ions was also found to be highly inhomogeneous in the methoxide-derived glass. All samples released soluble silica species on exposure to simulated body fluid, although only calcium-doped glasses exhibited *in vitro* bioactivity *via* the formation of hydroxyapatite. The biocompatibilities of model chitosan-glass GTR membranes were assessed using human MG63 osteosarcoma cells and were found to be of the order: CN < pure silica ≈ CC << CM ≈ CE. Calcium nitrate is the most commonly reported precursor for the sol-gel synthesis of bioactive glasses; however, the incomplete removal of nitrate ions during washing compromised the cytocompatibility of the resulting glass. The superior bioactivity and biocompatibility of the alkoxide-derived glasses is attributed to their ease of dissolution and lack of residual toxic anions. Overall, calcium ethoxide was found to be the preferred precursor with respect to extent of calcium-incorporation, homogeneity, bioactivity and biocompatibility.

## 1. Introduction

Periodontitis is a widespread infectious disease characterised by inflammation and progressive destruction of the tooth attachment apparatus [1,2,3]. Guided tissue regeneration (GTR) of damaged periodontal structures involves the placement of a barrier membrane to exclude the fast-growing epithelial tissues from the exposed root surface in order to enable the more slow-growing ligament and hard tissues to regenerate [1,2,3]. GTR membranes have been engineered from a number of resorbable biocompatible polymers, including both synthetic (e.g., poly(lactic acid) and poly(caprolactone)) and natural (e.g., chitosan, alginate and collagen) materials [1,2,3,4]. Various silica-based phases, such as multi-component glasses [2], Stöber particles [5], minerals [6] and diatomite [7] have been incorporated into the polymer matrices in order to enhance the mechanical and/or osteogenic properties of the GTR membranes. 

The Stöber process is a simple, ambient-temperature, one-pot, sol-gel processing route for the synthesis of spherical monodispersed silica (SiO_2_) particles (between 0.05 and 2 μm in diameter) [8]. Traditionally, Stöber particles are prepared by ammonium hydroxide-catalysed hydrolysis and condensation of tetraethyl orthosilicate (TEOS) in aqueous ethanol at room temperature under vigorous stirring [8]. The original Stöber process has been widely modified to functionalise the resulting particles with fluorescent labels, therapeutic ions and a range of active pharmaceutical ingredients [9,10,11,12,13,14]. 

The research reported in the current paper was carried out to investigate the impact of different calcium reagents on the morphology, composition, bioactivity and biocompatibility of two-component (CaO-SiO_2_) glasses produced by the Stöber process with respect to their potential application in GTR membranes. The Stöber process was modified by the direct addition of calcium chloride, calcium nitrate, calcium methoxide or calcium ethoxide at 5 mol % with respect to TEOS in the reagent mixture. The composition and morphology of the glasses were characterised using powder X-ray diffraction analysis (XRD), Fourier transform infrared spectroscopy (FTIR), scanning electron microscopy (SEM) and energy dispersive X-ray analysis (EDX). The impact of the various calcium reagents on the bioactivity and dissolution behaviour of the glasses was assessed *in vitro* by immersion in simulated body fluid (SBF) for up to one week [15].

In order to evaluate biocompatibility, model composite GTR membranes incorporating the calcium-doped or pure silica Stöber particles were prepared by solvent-casting using chitosan as the polymer matrix. Chitosan is a biodegradable, biocompatible, haemostatic, linear co-polymer of glucosamine and N-acetylglucosamine whose structure resembles that of bone extracellular matrix (ECM) [6]. Chitosan-based GTR membranes have been shown to successfully promote the regeneration of the tooth attachment tissues and alveolar bone in canine and human trials [1,16]. The biocompatibilities of the chitosan-glass composites prepared in this study were assessed using human MG63 osteosarcoma cells *via* an MTT assay [6]. 

## 2. Results

### 2.1. Materials Characterisation

**Figure 1:**
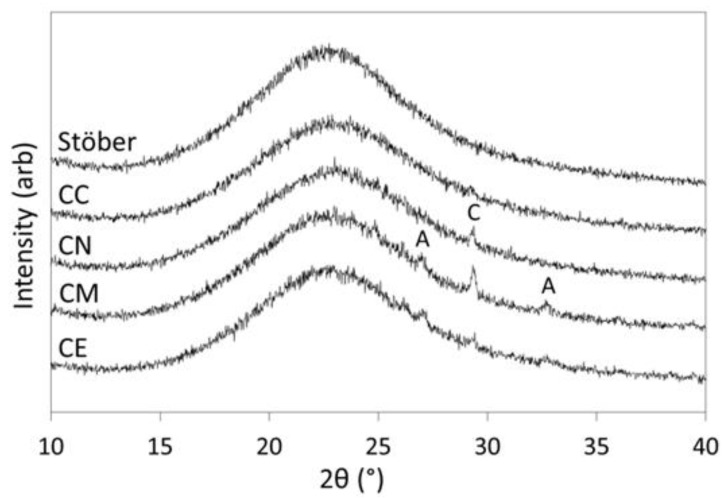
Powder XRD patterns of the Stöber sol-gel glass and those prepared with 5 mol % CaCl_2_ (CC), Ca(NO_3_)_2_ (CN), Ca(OMe)_2_ (CM) and Ca(OEt)_2_ (CE). Key: calcite—C and aragonite—A.

All dried glass samples presented as fine, white, free-flowing powders that were used without further modification or reduction in particle-size. Powder XRD patterns of the pure silica Stöber glass particles (*viz*. Stöber) and those prepared with 5 mol % calcium chloride (CC), calcium nitrate (CN), calcium methoxide (CM) or calcium ethoxide (CE) are presented in Figure 1. These data confirm the amorphous nature of the glasses produced by this method and indicate that trace quantities of calcium carbonate in the forms of calcite and aragonite are present, below 1 wt %, in the calcium-doped samples. Minor quantities of calcium carbonate commonly arise from atmospheric carbonation during sol-gel and hydrothermal preparations of calcium silicate phases under alkaline conditions [6]. In this initial study, sample preparations were carried out in air rather than under an inert atmosphere (e.g., nitrogen or argon gas) which could be used to preclude the formation of calcium carbonate but may give rise to calcium hydroxide. 

The molar Ca:Si ratios of the sol-gel glasses prepared with calcium chloride, calcium nitrate, calcium methoxide and calcium ethoxide were determined by EDX and are listed in Table 1. All Ca:Si ratios are found to be significantly lower than the nominal value of 0.05 demonstrating that, irrespective of the nature of the calcium precursor, the calcium ions are partitioned between the reaction liquor and the silicate network. The observed extent of incorporation of calcium into the glasses followed the sequence: ethoxide ≈ chloride > nitrate ≈ methoxide. The relative standard deviation (RSD) of the mean for the Ca:Si ratio of the glass prepared with calcium methoxide (CM) is 58% demonstrating that this sample has a highly inhomogeneous distribution of calcium at the micron-scale in comparison with the other samples whose RSD values do not exceed 8%. 

Only silicon and oxygen were detected in the EDX spectra of the pure silica Stöber particles, and no elements in addition to silicon, oxygen and calcium were detected in the calcium-doped glasses, indicating that the washing process removes residual chloride, nitrate and alkoxide species to below the limit of detection for this technique (~0.5 wt %). 

**Figure 2:**
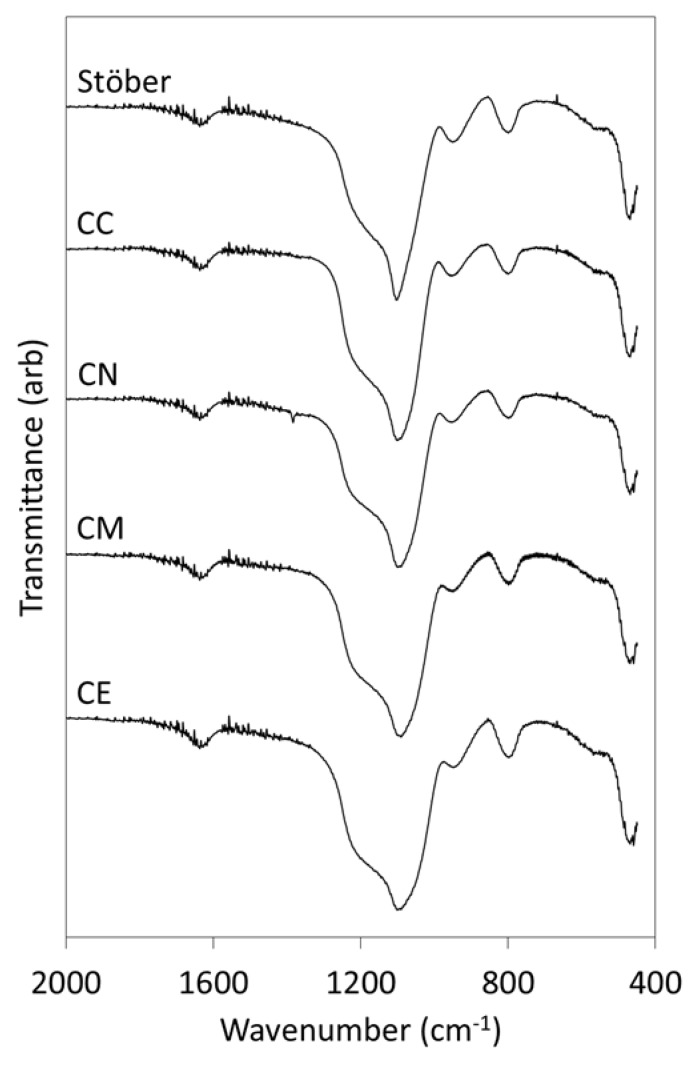
FTIR spectra of the Stöber sol-gel glass and those prepared with 5 mol % CaCl_2_ (CC), Ca(NO_3_)_2_ (CN), Ca(OMe)_2_ (CM) and Ca(OEt)_2_ (CE).

The FTIR spectra of the pure silica Stöber particles and those prepared with the various calcium precursors are presented in Figure 2. The assignments of the FTIR bands are given in Table 2 [10]. The scissor bending vibrations of adsorbed molecular water occur at approximately 1635 cm^−1^ and are similar for all samples. Various stretching modes of the Si-O-Si glass network give rise to the large combination band with maximum intensity *circa* 1100 cm^−1^. The maximum width of this signal (listed in Table 2) is seen to increase in the following order: Stöber < CC < CN < CE ≈ CM. The broader Si-O-Si bands observed for the calcium-doped glasses denote increasing disorganisation and decreasing polymerisation within the glass network arising from the incorporation of the network modifying Ca^2+^ ions [17]. The asymmetric stretching modes of the Si-OH groups that occur at 950 cm^−1^ and the symmetric Si-O vibrations at ~800 cm^−1^ appear unaffected by the incorporation of Ca^2+^ ions. 

The antisymmetric stretching vibration of the nitrate ion is visible at 1390 cm^−1^ in the FTIR spectrum of the calcium-doped glass derived from the nitrate precursor (CN) [18], which demonstrates that this ion was not completely removed by washing. Vibrations from calcium carbonate (a trace impurity that was observed in the calcium-doped samples by XRD analysis, Figure 1) are not apparent in the FTIR spectra of any of the glasses, indicating that this phase was present below the limit of detection for this technique. 

**Figure 3:**
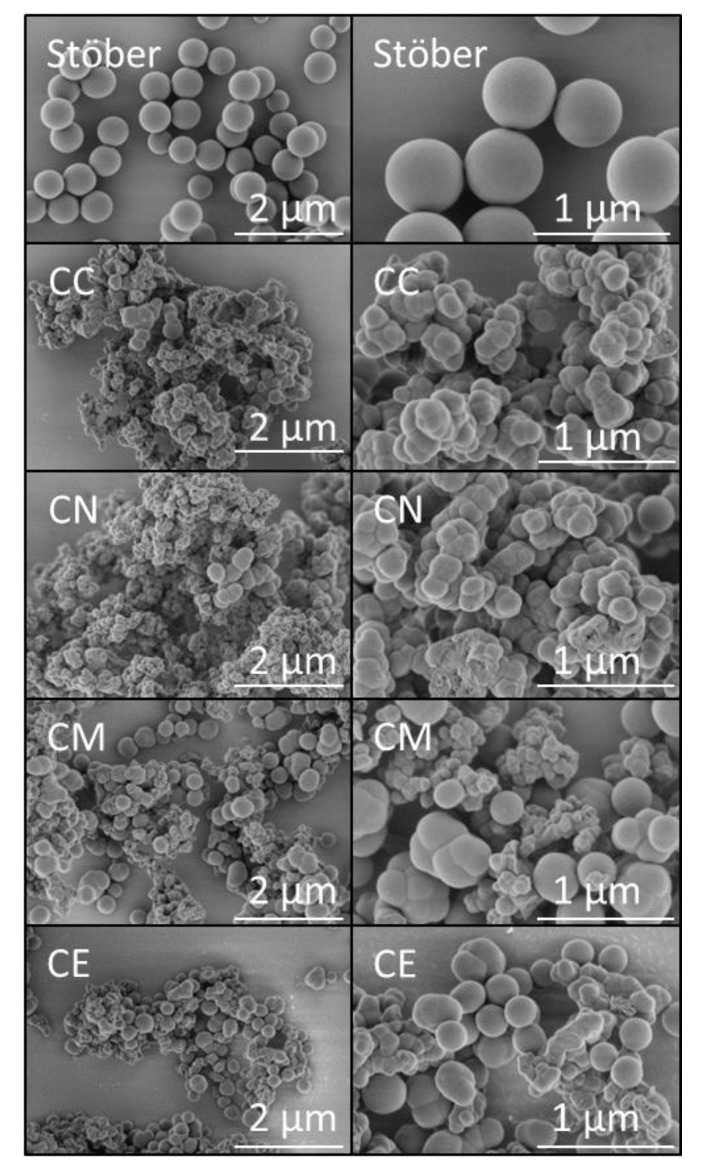
SEM images of the Stöber sol-gel glass and those prepared with 5 mol % CaCl_2_ (CC), Ca(NO_3_)_2_ (CN), Ca(OMe)_2_ (CM) and Ca(OEt)_2_ (CE) at ×20k (**left**) and ×50k (**right**) magnifications.

Secondary electron SEM images of the uncoated pure silica Stöber particles and the calcium-doped glasses are shown in Figure 3. The pure silica particles typify those produced by the traditional Stöber process under the selected reaction conditions, as they are smooth, spherical and monodispersed with a diameter of ~500 nm [8]. In all cases, the addition of 5 mol % calcium precursor to the reaction mixture resulted in highly textured micron-sized agglomerated clusters of nanoparticles. The chloride and nitrate precursors gave rise to 2–20 μm diameter aggregates of irregularly shaped globular nanoparticles and the calcium alkoxides produced 1–5 μm clusters of globules and distorted nanospheres.

### 2.2. In Vitro Bioactivity and Dissolution Characteristics

Bioactive materials bond to living bone tissue *via* a layer of substituted hydroxyapatite (HA) that precipitates spontaneously at the surface on implantation in the body [15]. This HA-layer provides a focus for the attachment and proliferation of osteocytes thereby stimulating bone tissue regeneration. A semiquantitative measure of bioactivity can be obtained by monitoring the formation of a substituted hydroxyapatite layer on the surface of a material immersed in SBF [15]. 

**Figure 4:**
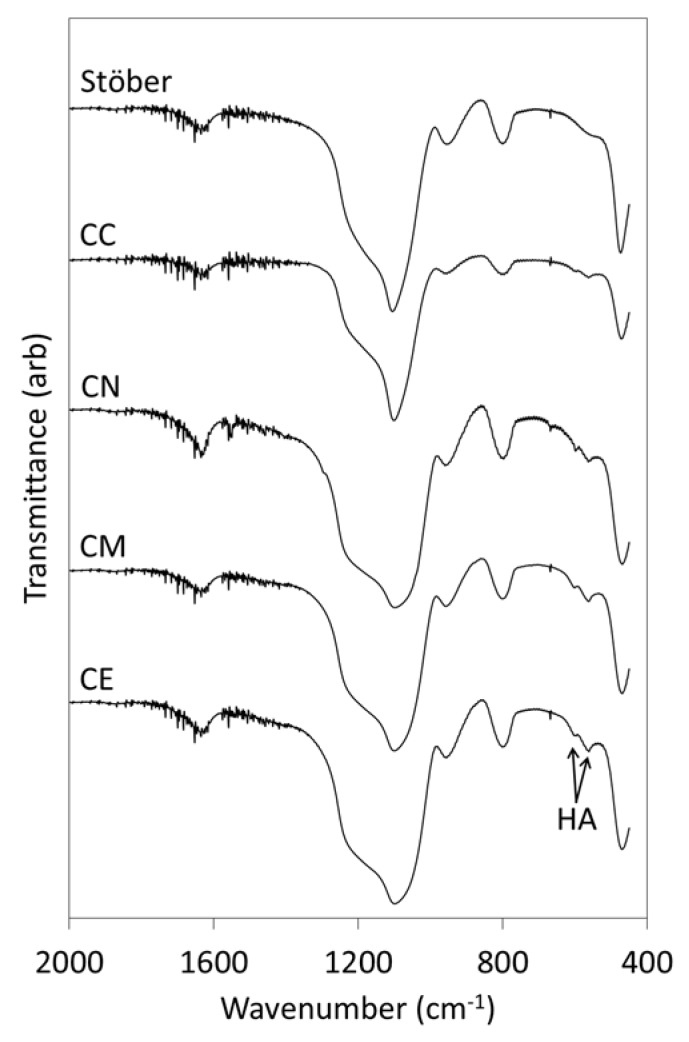
FTIR spectra of the Stöber sol-gel glass and those prepared with 5 mol % CaCl_2_ (CC), Ca(NO_3_)_2_ (CN), Ca(OMe)_2_ (CM) and Ca(OEt)_2_ (CE) following immersion in SBF for 1 week.

**Figure 5:**
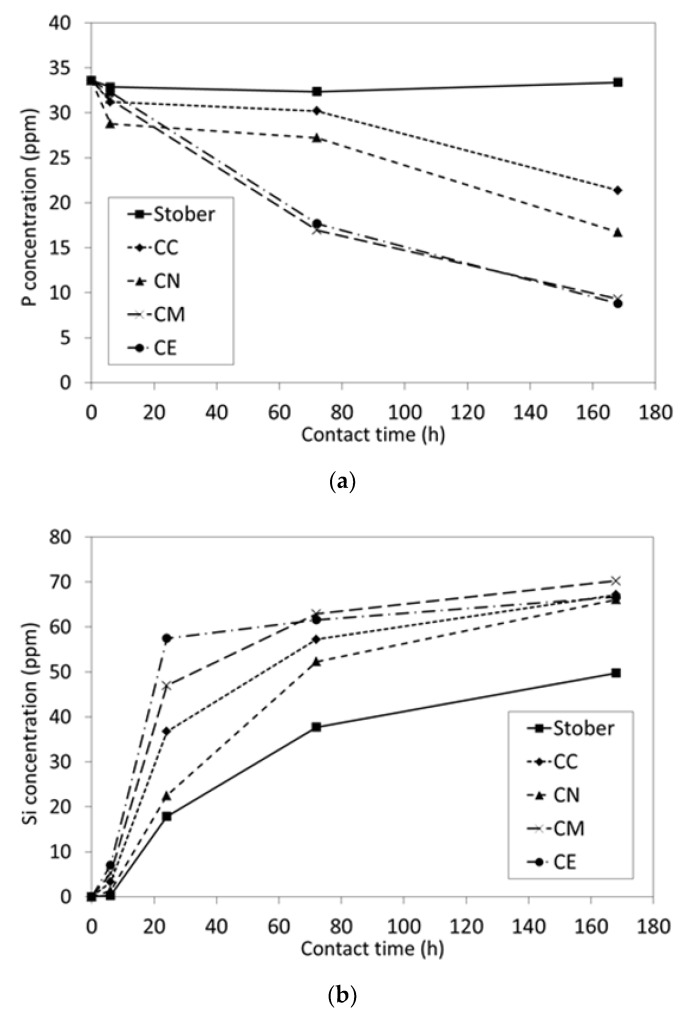
SBF concentration profiles of (**a**) P and (**b**) Si for Stöber sol-gel glass and those prepared with 5 mol % CaCl_2_ (CC), Ca(NO_3_)_2_ (CN), Ca(OMe)_2_ (CM) and Ca(OEt)_2_ (CE) as functions of time.

The FTIR spectra of the glass samples following exposure to SBF for 1 week are presented in Figure 4 and the corresponding SBF-concentration profiles for P and Si are plotted in Figure 5. 

The FTIR spectrum of pure silica Stöber particles after exposure to SBF for 1 week (Figure 4) does not differ significantly from that of the original sample (Figure 2) which indicates that this material does not elicit the nucleation and growth of HA. The concomitant concentration of phosphate species in contact with the pure silica sample (Figure 5a) does not change significantly throughout this timeframe which confirms the lack of bioactivity of this material.

Conversely, evidence for the formation of HA on the surfaces of all of the calcium-doped glasses following immersion in SBF is denoted by the doublet at 570 and 605 cm^−1^ that is characteristic of the P-O bending modes in crystalline HA (Figure 4) [12,17]. The relative intensities of the HA bands are seen to increase in the following order: CC < CN < CM ≈ CE. The phosphate ion profiles of the supernatant SBF solutions in contact with the calcium-doped samples (Figure 5a) are consistent with the FTIR data and also indicate that the extents of removal of phosphate ions by the alkoxide-derived glasses are greater than those of their nitrate- and chloride-derived counterparts with the latter exhibiting the slowest rate of HA-precipitation.

The release of soluble silica from the pure silica Stöber particles and calcium-doped glasses, plotted in Figure 5b, show that the initial dissolution rates are of the following order: CE > CM > CC > CN > Stöber. The dissolution rates of silica species from binary calcium silicate glasses are governed, *inter alia*, by calcium-content and the degree of polymerisation of the silicate network [12,17,19]. Initially, dissolution proceeds *via* cation exchange between Ca^2+^ ions in the glass and H^+^ ions from SBF which facilitates the release of soluble silica. Hence, superior solubility is generally observed for increasing Ca/Si ratios. Likewise, poorly polymerised glasses with low silicate network connectivity are also more soluble in aqueous media. 

### 2.3. In Vitro Cytocompatibility of Composite Chitosan-Glass Membranes

**Figure 6:**
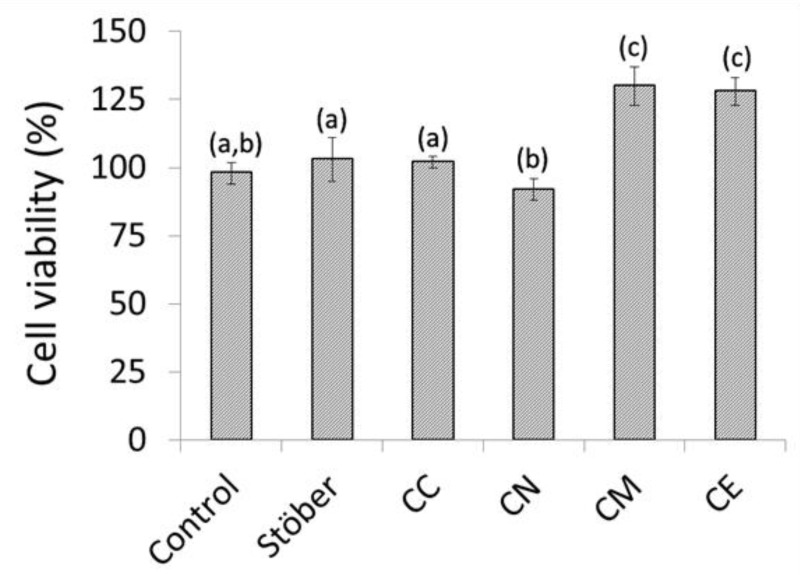
In vitro cytocompatibilities of Stöber sol-gel glass and those prepared with 5 mol % CaCl_2_ (CC), Ca(NO_3_)_2_ (CN), Ca(OMe)_2_ (CM) and Ca(OEt)_2_ (CE) incorporated into chitosan membranes. Different superscript letters indicate significant differences between the groups and the same letters indicate no significant differences (*p* = 0.05).

Human osteosarcoma cells are a popular *in vitro* model for the initial cytocompatibility assessment of implantable biomaterials for dental and orthopaedic applications [6,20,21,22,23,24,25]. In this study, the indirect cytotoxicity of composite chitosan-glass membranes towards MG63 human osteosarcoma cells was evaluated using an MTT assay [6,24]. MG63 osteosarcoma cells are easily cultured, exhibit many of the features of the pre-osteoblastic stage and maintain high phenotypic stability over serial passages [25]. Cell viability data for MG63 osteosarcoma cells cultured in contact with pure chitosan (Control) and composite chitosan-glass membranes are expressed relative to that of the culture consisting of cells and medium only in Figure 6. These data show no significant differences in cell viability among the cultures in contact with the pure chitosan control and the composites incorporating either Stöber particles or the calcium chloride-derived glass (*p* = 0.05, n = 4). A modest reduction in cytocompatibility is observed for the membrane containing the calcium nitrate-derived glass, whereas cell viabilities are seen to increase significantly for the cultures in contact with the alkoxide-derived glasses. In fact, the presence of the CM and CE membranes was found to enhance cell growth by 33% and 31%, respectively, relative to that of the pure chitosan control. 

## 3. Discussion

### 3.1. Pure Silica Stöber Particles

Silica Stöber particles are traditionally prepared by ammonium hydroxide-catalysed hydrolysis and condensation of TEOS in aqueous ethanol at room temperature [8]. Spherical monodispersed particles are obtained at molar water:TEOS ratios greater than 20 and at pH values above 10 units [8,26]. The high water concentration and alkalinity both accelerate the condensation reactions that give rise to compact silica clusters rather than weakly cross-linked extended networks associated with acid catalysis. The Stöber reaction is characterised by rapid nucleation followed by isotropic aggregation of smaller particles in a mechanism similar to that of coacervation [26]. Under the highly basic conditions, that far exceed the isoelectric point of silica (pH~2), negative electrostatic repulsive forces prevent the agglomeration and coalescence of the evolving particles. 

The spherical monodispersed ~500 nm pure silica particles obtained in this study are typical of those produced by the traditional Stöber process under the selected reaction conditions (water:TEOS = 51, initial pH~11.7) [8]. These Stöber particles did not exhibit *in vitro* bioactivity (Figure 4) and were found to have no significant impact on biocompatibility when incorporated in a model chitosan GTR membrane (Figure 6). With very few exceptions [27], pure silica gels and glasses do not exhibit *in vitro* bioactivity *via* the formation of a layer of hydroxyapatite on immersion in SBF. Bioactivity is generally attributed to materials that release calcium ions to promote local supersaturation with respect to hydroxyapatite and that concomitantly present a hydrated silica gel layer comprising surface silanol (≡Si-OH) groups that act as nucleation sites [10,15,28,29]. 

In a recent study, pure silica Stöber particles have been shown to improve the mechanical properties of poly(caprolactone)-based composite GTR membranes without compromising cytocompatibility *in vitro* [5]. Despite their intrinsic lack of *in vitro* bioactivity, the observed release of soluble silica species (Figure 5b), which are known to enhance osteogenesis, may confer additional therapeutic effects *in vivo* [28]. Furthermore, the ambient temperature processing route afforded by the Stöber process also offers the potential for functionalisation with organic and biological payloads such as growth factors [30]. In this respect, bone marrow-derived stem cells have been shown to internalise ~215 nm Stöber particles, which demonstrates their potential as vectors for the direct and sustained delivery of active agents [31]. 

### 3.2. Calcium-Doped Glasses

The acid-catalysed sol-gel synthesis of two-component calcium silicate bioactive glasses from TEOS and various calcium-bearing reagents is widely reported in the literature [12,17,19]. Under acidic conditions, the hydrolysis and condensation reactions of TEOS result in a loosely cross-linked silica network that collapses on drying to form a nanoporous monolithic gel. The product can then be subjected to thermal treatment to remove or reduce the concentration of surface silanol groups and to densify the glass. Small components of bespoke geometry can be prepared by this method if shrinkage is taken into account; however, most commonly the glass product is mechanically ground, sieved and graded prior to use in particulate form. 

To date, calcium nitrate is the most frequently employed calcium precursor for the preparation of sol-gel-derived bioactive glasses [19,32]. Under acidic conditions, the soluble Ca^2+^ and NO_3_^−^ ions remain dissolved in the pore liquor as the gel forms and are not directly incorporated into the silicate network. On drying, non-uniform precipitates of calcium nitrate are deposited onto the surface of the gel with calcium-rich regions at the edges. The calcium ions are subsequently incorporated into the glass as network modifiers by heating above 400 °C and the toxic nitrate ions are removed by thermal decomposition above 500 °C. Hence, the principal limitations of calcium nitrate as a reagent for the acid catalysed sol-gel synthesis of calcium silicate glasses are that the distribution of Ca^2+^ ions throughout the silicate network is inhomogeneous and the high processing temperatures preclude the incorporation of potentially beneficial organic components [19]. 

Calcium chloride has also proven to be a poor alternative to the nitrate precursor, as Ca^2+^ ions derived from the chloride salt fail to enter the silicate network at temperatures up to 800 °C [19]. Instead an unidentified crystalline phase is found to precipitate on heating to 700 °C. In addition, though not essentially toxic, the fate of the chloride ions in these glasses as a function of temperature is currently unknown. Similarly, calcium acetate is an unsuitable precursor as it visibly precipitates during drying [19] and a temperature of 600 °C is required to integrate Ca^2+^ ions into the silicate network if calcium hydroxide is used [33]. 

Conversely, under acid catalysis, calcium methoxyethoxide is reported to hydrolyse and condense together with TEOS thus becoming incorporated into the silicate network at room temperature [19]. In this respect, calcium methoxyethoxide is recommended in preference to the other common salts of calcium for superior homogeneity and for the ambient temperature synthesis of glass-organic hybrids. 

Literature reports of ambient temperature, non-templated, base-catalysed Stöber syntheses of bioactive calcium silicate glasses are rare [10]. Several investigations that exploit surfactants and structure-directing agents or involve the *post hoc* incorporation of Ca^2+^ ions into Stöber particles are described [12,34,35]; however, these processes all involve high finishing temperatures (≥600 °C) in order to integrate the Ca^2+^ ions into the glass and to decompose unwanted organics and/or nitrates. 

Chen et al. [10] produced spherical calcium silicate particles by the Stöber process *via* the direct addition of calcium chloride at molar CaCl_2_:TEOS ratios of 0.0041 and 0.0082. EDX and dissolution analyses confirmed the presence of Ca^2+^ ions within the silicate network; although, at these low Ca:Si ratios, no significant differences were noted in the FTIR or ^29^Si MAS NMR spectra between the pure silica and Ca-doped glasses. The Ca-doped glasses were larger than their pure silica counterparts with some inter-particle necking which was attributed to increased aggregation arising from the presence of the calcium cations. Similarly to the findings of the present study, the Ca-doped glasses exhibited *in vitro* bioactivity whereas the pure silica particles did not, indicating that the presentation of pre-existing silanol groups is not sufficient for the precipitation of hydroxyapatite in the absence of leachable Ca^2+^ ions. 

In our study, irrespective of the nature of the calcium precursor, the higher nominal Ca:Si ratio of 0.05 resulted in significant agglomeration of the glass particles, which gave rise to nano-textured, micron-sized aggregates rather than monodispersed spheres (Figure 3). The presence of the Ca^2+^ ions in the reaction mixture reduced the negative electrostatic repulsive forces generated by the high basicity and caused the evolving particles to coalesce. Accordingly, chloride and nitrate precursors gave rise to 2–20 μm diameter aggregates and the calcium alkoxides produced aggregates in the 1–5 μm range. Hence, the direct addition of the calcium precursors to the Stöber process markedly reduced control of particle size. However, unlike glasses prepared *via* acid catalysis, the Ca-doped glass particles produced by this method did not require grinding or sieving prior to use (i.e., incorporation into the chitosan membranes). Additionally, it is considered that their nano-textured surfaces may also be favourable for osteocyte-attachment and mineralisation [36,37]. 

The broadening of the Si-O-Si band at ~1100 cm^−1^ in the infrared spectra of the Ca-doped glasses in this study demonstrated that the silicate network was disrupted by the incorporation of the network modifying Ca^2+^ ions (Figure 2). EDX analysis confirmed that, in all cases, the Ca:Si ratios were significantly lower than the nominal value of 0.05 (Table 1), and that the distribution of calcium within the methoxide-derived glass was highly inhomogeneous, although the reason for this is not known. 

In contrast to the acid-catalysed methods reported in the literature [12,17,19], the successful incorporation of Ca^2+^ ions into the silicate network from various calcium precursors at ambient temperature, observed by Chen et al. [10] and in the current study, is attributed to the high pH of the reaction medium. Under the highly basic conditions associated with the Stöber process, the condensation reaction is promoted over hydrolysis and involves the nucleophilic attack of deprotonated silanol (≡SiO^−^) species [26]. The free Ca^2+^ ions in the reaction liquor electrostatically interact with these anionic ≡SiO^−^ intermediates and are thus taken up into the silicate network. Under acidic conditions, hydrolysis proceeds *via* the rapid protonation of the TEOS molecule and the subsequent slow condensation reactions involve protonated silanol species which do not readily interact with the free calcium cations in solution. 

One distinct advantage of the direct incorporation of Ca^2+^ ions into the silicate network under basic conditions is that this method potentially allows for the removal of residual supernatant Ca^2+^ ions and undesirable contaminant anions (e.g., Cl^−^, NO_3_^−^, CH_3_O^−^ and CH_3_CH_2_O^−^) by washing with water. This eliminates the need for high temperature processing; although, in our study the removal of nitrate ions was incomplete which compromised the cytocompatibility of the resulting glass. Conversely, although with the possible exception of calcium methoxyethoxide [19], it is not feasible to wash unwanted soluble species from calcium silicate gels prepared under acidic conditions as this would also cause the removal of the Ca^2+^ ions. Presumably, the nanoporous and monolithic nature of the acid-catalysed gels would also present a significant challenge for the successful elimination of contaminants from within the pore system by washing. 

As is commonly the case during sol-gel and hydrothermal preparations of calcium silicate phases under alkaline conditions, trace quantities (<1 wt %) of calcium carbonate in the forms of calcite and aragonite were present in the calcium-doped samples (Figure 1) [6,24,34]. All crystalline polymorphs of calcium carbonate are biocompatible with respect to bone tissue, and in fact, amorphous calcium carbonate acts as a ‘bioseed’ precursor for the formation of hydroxyapatite in human bone [38]. Accordingly, the presence of minor quantities of calcium carbonate would not be detrimental to the biocompatibility of a glass in periodontal applications, but it does represent limitations on the reproducibility and compositional range of calcium silicate glasses that can be produced under alkaline conditions. 

Calcium hydroxide is only sparingly soluble in water (K_sp_~5.02 × 10^−6^) and its solubility dramatically decreases with increasing pH [39]. This common ion effect limits the maximum concentration of dissolved Ca^2+^ ions as a function of the concentration of ammonia (or any other base) in aqueous media. The maximum concentration of base that could be used without causing the precipitation of calcium hydroxide depends on the nature and concentration of the calcium precursor and the other components in the modified Stöber reaction mixture. Above this threshold base concentration, the precipitation of calcium hydroxide is inevitable; although, an inert atmosphere could be used to preclude the formation of calcium carbonate at concentrations below this value. In comparison with the acid-catalysed sol-gel methods, this phenomenon imposes a considerable restriction on the compositional ranges of binary and multicomponent calcium silicate glasses that are achievable under alkaline conditions. 

The low concentrations of calcium carbonate in the Ca-doped glasses in this study are not considered to have had any significant impact on the *in vitro* bioactivity or biocompatibility of these materials. All Ca-doped glasses effected the precipitation of crystalline hydroxyapatite following immersion in SBF for 1 week (Figure 4). The rates of dissolution of silica species were found to be greatest for the calcium alkoxide-derived glasses which correlated with the highest observed rates of precipitation of hydroxyapatite (Figure 5). These alkoxide-derived glasses also exhibited superior biocompatibility when incorporated into model chitosan GTR membranes. 

Chitosan is a popular candidate membrane material for periodontal repair owing to its biocompatibility with both hard and soft dental tissues, biodegradability and similarity in structure to bone ECM; however, pure chitosan is insufficiently bioactive in this application and its acidic degradation products are reported to provoke inflammatory reactions which inhibit healing [40,41]. A wide range of inorganic phases including, synthetic hydroxyapatite, β-tricalcium phosphate, silica, and bioactive glasses have been incorporated into chitosan-based composites to improve bioactivity [1,3,33,40,41,42,43]. In addition to enhanced osteogenesis, advantages of using calcium silicate glasses in chitosan-based GTR applications are that both materials are biodegradable which negates the need for second surgery to remove the membrane, the alkaline breakdown products of the glass are able to buffer the acidic degradation products of chitosan and the glass particles will generally confer enhanced mechanical properties. 

The results of the cytotoxicity assay (Figure 6) support those of the *in vitro* bioactivity evaluation and confirm that the growth of human osteoblastic cells was enhanced by 33% and 31% in the presence of the chitosan membranes blended with the calcium methoxide- and ethoxide-derived glasses, respectively. Calcium nitrate is currently the most commonly reported precursor for the sol-gel synthesis of bioactive glasses; however, the incomplete removal of nitrate ions during washing is thought to have compromised the cytocompatibility of the resulting glass.

The superior bioactivity and biocompatibility of the alkoxide-derived glasses observed during this investigation are attributed to their ease of dissolution and lack of residual toxic anions. Of the calcium reagents considered in this study, calcium ethoxide is the preferred precursor with respect to extent of calcium-incorporation, homogeneity, bioactivity and biocompatibility.

During the next phase of this research, the formulation of composite GTR membranes of chitosan and calcium ethoxide-derived glasses will be optimised for their mechanical strength and degradation characteristics [43]. Selected candidate composite membranes will then be appraised for their impact on the proliferation and expression of osteocyte, human bone marrow, ligament and cementoblast cell lines [3,44,45,46]. 

## 4. Materials and Methods 

All reagents, with the exception of calcium ethoxide (Ca(OC_2_H_5_)_2_), were purchased from Sigma-Aldrich (Gillingham, UK). Ammonium hydroxide (ACS reagent, 30% NH_3_), tetraethyl orthosilicate (TEOS, reagent grade, 98%), calcium chloride dihydrate (CaCl_2_.2H_2_O, ACS reagent, ≥99%), calcium nitrate tetrahydrate (Ca(NO_3_)_2_.4H_2_O, ACS reagent, 99%), calcium methoxide (Ca(OCH_3_)_2_, 97%), calcium metal (granular, 99%) and chitosan (50–190 kDa molecular weight, 75–85% deacetylated) were used without further modification or purification. Absolute ethanol (ACS reagent, 200 proof, ≥99.5%) was dehydrated using a 4 Å molecular sieve prior to the preparation of calcium ethoxide. 

Calcium ethoxide was prepared by direct reaction of calcium metal with excess dehydrated ethanol (reaction ratio 1:40) by boiling under reflux in an argon atmosphere with stirring for 3 h [47]. The solid white product was isolated by distillation of the supernatant ethanol and confirmed as calcium ethoxide by FTIR.

### 4.1. Preparation and Characterisation of Glasses

To prepare the pure silica Stöber particles, a solution containing 2.23 cm^3^ of TEOS and 17.9 cm^3^ of ethanol was added to a solution containing 17.9 cm^3^ of ethanol, 9.1 cm^3^ of deionised water and 2.9 cm^3^ of 30% ammonium hydroxide under vigorous stirring. The mixture was stirred for 2 h and aged at 25 °C for 48 h. The two-component (CaO-SiO_2_) glasses were prepared similarly by the addition of a solution containing 2.23 cm^3^ of TEOS, 0.5 mmol of the relevant calcium reagent and 17.9 cm^3^ of ethanol to the receiving solution of ammonium hydroxide in aqueous ethanol, as described above. After aging, the samples were centrifuged and washed with three 20 cm^3^ aliquots of deionised water prior to drying at 80 °C in air to constant mass. Each synthesis was carried out in triplicate. The sample names and reaction compositions are listed in Table 3. 

The Stöber particles and two-component glass products were analysed by powder XRD using a Bruker D8 diffractometer (Bruker AXS, Karlsruhe, Germany) with Cu Kα = 1.5406 Å, a step size of 0.019° in the 2θ range from 5 to 40° and a measuring time of 1 s per step. FTIR spectra were acquired using a Perkin Elmer Spectrum Two spectrometer (Perkin Elmer, London, UK) between 450 and 2000 cm^−1^ wavenumbers, with 10 scans at a resolution of 4 cm^−1^. Secondary electron images of the products were obtained from uncoated samples attached to carbon tabs on a Hitachi SU8030 scanning electron microscope (Hitachi High-Technologies, Tokyo, Japan) with an accelerating voltage of 0.5 kV. EDX data were collected in triplicate from areas of 1 μm^2^ from compacted samples using a JEOL JSM-5410 LV electron microscope (JEOL, Tokyo, Japan) with an Oxford Instruments X-MaxN EDX detector (Oxford Instruments, Abingdon, UK) in low vacuum mode with an accelerating voltage of 8 kV. 

### 4.2. In Vitro Bioactivity Analysis

Simulated body fluid (SBF) was prepared in accordance with the method described in reference 15 and stored in polypropylene bottles at 4 °C for no longer than 3 days. The glass specimens were immersed in SBF (at a solid:solution ratio of 1.0 mg cm^−3^) in hermetically-sealed polypropylene containers at 37 °C for 6, 24, 72 and 168 h. Each analysis was carried out in triplicate. Solution concentrations of phosphorus and silicon were monitored as functions of time by inductively coupled plasma analysis (ICP) using a TJA Iris simultaneous ICP-OES spectrophotometer (Thermo Fisher Scientific, East Grinstead, UK) and matrix-matched multi-element standards. Relative standard deviations of the mean concentrations of phosphorous and silicon were below 10% in all cases. The glass particles were recovered from SBF solution by centrifugation, washed with deionised water and dried in air at 37 °C prior to analysis by FTIR. 

### 4.3. In Vitro Cytocompatibility Study

Chitosan-glass composite membranes were prepared in triplicate by dissolving 1 wt % chitosan in 2 wt % aqueous acetic acid solution and adding the glass powder at a chitosan:glass mass ratio of 1.5 under stirring. The solutions were cast on to polycarbonate surfaces and dried to constant mass in air at 60 °C [6]. A control membrane of pure chitosan was also prepared similarly without the addition of glass. The *in vitro* biocompatibilities of the control and chitosan-glass membranes were evaluated using MG63 human osteosarcoma cells (ECACC code: 86051601) as described in reference 4. In quadruplicate, one section of control or chitosan-glass composite membrane (1 mm × 4 mm) was incubated in a 96-well plate with 0.2 cm^3^ culture of MG63 cells (10^6^ cells cm^−3^) for 24 h and cell viability was established using an MTT (3-(4,5-dimethyl-2-thiazolyl)-2,5-diphenyl-2H-tetrazolium bromide) assay. UV-vis absorbance data were collected at 540 nm using a Multiskan Ascent microplate photometer plate reader (Thermo Fisher Scientific, East Grinstead, UK). Absorbance data for the control and chitosan-glass membranes were expressed as percentages relative to that of a culture containing only cells and subjected to a one-tailed *t*-test at (n–2) degrees of freedom and *p* = 0.05. 

## 5. Conclusions 

Binary and multicomponent sol-gel-derived calcium silicate glasses are most commonly produced by the acid-catalysed condensation of tetraethyl orthosilicate (TEOS) in the presence of calcium nitrate. Under these conditions, calcium ions do not enter the glass network below 400 °C and the cytotoxic nitrate ions require removal by thermal decomposition above 500 °C. Similarly, temperatures in excess of 600 °C are required to integrate calcium ions into the silicate network if calcium hydroxide reagent is used, and chloride-derived calcium ions fail to enter the silicate network below the point of crystallisation at ~700 °C. Calcium methoxyethoxide (CME) is currently the only calcium precursor that has been used to successfully integrate calcium ions into the glass network at room temperature. 

This study investigated the impact of different calcium reagents on the morphology, composition, bioactivity and biocompatibility of binary (CaO-SiO_2_) glasses produced by the base-catalysed Stöber process. The properties of the binary glasses were compared with those of pure silica Stöber particles. Under the selected conditions (water:TEOS = 51, initial pH ~11.7), calcium ions derived from 5 mol % addition of calcium chloride (CC), calcium nitrate (CN), calcium methoxide (CM) or calcium ethoxide (CE) were found to enter the silicate network at room temperature, albeit at concentrations below the nominal Ca/Si ratio of 0.05. The distribution of calcium ions was found to be highly inhomogeneous in the methoxide-derived glass. 

All samples released soluble silica species on exposure to simulated body fluid, although only calcium-doped glasses exhibited *in vitro* bioactivity *via* the formation of hydroxyapatite. The biocompatibilities of model chitosan-glass composites were assessed using human MG63 osteosarcoma cells and the superior biocompatibility of the alkoxide-derived glasses was attributed to their ease of dissolution and lack of residual toxic anions. With respect to the extent of calcium-incorporation, homogeneity, bioactivity and biocompatibility, calcium ethoxide was found to be the preferred precursor. 

The ambient temperature processing route afforded by this base-catalysed modified Stöber process offers the potential for the production of sol-gel hybrids and the functionalisation of the glasses with therapeutic organic and biological components that cannot be achieved at elevated temperatures. 

## Figures and Tables

**Table 1:** EDX data for molar Ca:Si ratios of Stöber sol-gel glass and those prepared with 5 mol % CaCl_2_ (CC), Ca(NO_3_)_2_ (CN), Ca(OMe)_2_ (CM) and Ca(OEt)_2_ (CE).

Sol-Gel Glass	Molar Ca:Si Ratio ^1^
Ideal glass with 5 mol % Ca^2+^	0.05
Stöber	0.00 ^a^
CC	0.036 ± 0.002 ^b^
CN	0.029 ± 0.002 ^c^
CM	0.024 ± 0.014 ^b,c^
CE	0.037 ± 0.003 ^b^

^1^ Different superscript letters indicate significant differences between the groups and the same letters indicate no significant differences (*p* = 0.05).

**Table 2:** FTIR assignments of major bands of Stöber particles and calcium-doped glasses [10].

Sol-Gel Glass	H_2_O Bend (cm^−1^)	Si-O-Si Network (cm^−1^)	Max. width Si-O-Si Band (cm^−1^)	Si-OH asym str (cm^−1^)	Si-O sym (cm^−1^)
Stöber	1638	1104	310	950	799
CC	1631	1099	320	951	799
CN	1636	1098	330	951	802
CM	1631	1089	363	950	798
CE	1638	1092	360	947	799

**Table 3:** Reaction compositions of Stöber particles and two-component (CaO-SiO_2_) glasses.

Sample Name	Calcium Reagent	Mass of Calcium Reagent (mg)	Molar Ratio Ca:Si
Stöber	–	–	0.00
CC	CaCl_2_·2H_2_O	73.5	0.05
CN	Ca(NO_3_)_2_·4H_2_O	118.1	0.05
CM	Ca(OCH_3_)_2_	51.1	0.05
CE	Ca(OC_2_H_5_)_2_	65.1	0.05

## References

[1] Vaca-Cornejo F., Macías Reyes H., Dueñas Jiménez S.H., Llamas Velázquez R.A., Dueñas Jiménez J.M. (2017). Pilot study using a chitosan-hydroxyapatite implant for guided alveolar bone growth in patients with chronic periodontitis. J. Funct. Biomater..

[2] Dehnavi S.S., Mehdikhani M., Rafienia M., Bonakdar S. (2018). Preparation and in vitro evaluation of polycaprolactone/PEG/bioactive glass nanopowders nanocomposite membranes for GTR/GBR applications. Mater. Sci. Eng. C.

[3] Mota J., Yuc N., Caridade S.G., Luz G.M., Gomes M.E., Reis R.L., Jansen J.A., Walboomers F., Mano J.F. (2012). Chitosan/bioactive glass nanoparticle composite membranes for periodontal regeneration. Acta Biomater..

[4] Srinivasan S., Jayasree R., Chennazhi K.P., Nair S.V., Jayakumar R. (2012). Biocompatible alginate/nano bioactive glass ceramic composite scaffolds for periodontal tissue regeneration. Carbohydr. Polym..

[5] Castro A.G.B., Diba M., Kersten M., Jansen J.A., van den Beucken J.J.J.P., Yang F. (2018). Development of a PCL-silica nanoparticles composite membrane for guided bone regeneration. Mater. Sci. Eng. C.

[6] Hurt A.P., Getti G., Coleman N.J. (2014). Bioactivity and biocompatibility of a chitosan-tobermorite composite membrane for guided tissue regeneration. Int. J. Biol. Macromol..

[7] Tamburaci S., Tihminlioglu F. (2017). Diatomite reinforced chitosan composite membrane as potential scaffold for guided bone regeneration. Mater. Sci. Eng. C.

[8] Stöber W., Fink A., Bohn E. (1968). Controlled growth of monodisperse silica spheres in micron size range. J. Colloid Interface Sci..

[9] Riccò R., Nizzero S., Penna E., Meneghello A., Cretaio E., Enrichi F. (2018). Ultra-small dye-doped silica nanoparticles via modified sol-gel technique. J. Nanopart. Res..

[10] Chen S., Osaka A., Hayakawa S., Kanji Tsuru K., Fujii E., Kawabata K. (2008). Microstructure evolution in Stöber-type silica nanoparticles and their in vitro apatite deposition. J. Sol-Gel. Sci. Technol..

[11] Rocha de Oliveira A.A., de Souza D.A., Silveira Dias L.L., de Carvalho S.M., Mansur H.S., Pereira M.d.M. (2013). Synthesis, characterization and cytocompatibility of spherical bioactive glass nanoparticles for potential hard tissue engineering applications. Biomed. Mater..

[12] Greasley S.L., Page S.J., Sirovica S., Chen S., Martin R.A., Riveiro A., Hanna J.V., Porter A.E., Jones J.R. (2016). Controlling particle size in the Stöber process and incorporation of calcium. J. Colloid Interface Sci..

[13] Kim Y.H., Lee D.K., Kim C.W., Cha H.G., Kang Y.S., Jo B.G., Jeong J.H. (2007). Preparation and antibiotic property of Ag-SiO_2_ nanoparticle. Mol. Cryst. Liq. Cryst..

[14] Gonçalves M.C. (2018). Sol-gel silica nanoparticles in medicine: A natural choice. Design, synthesis and products. Molecules.

[15] Kokubo T., Takadama H. (2006). How useful is SBF in predicting in vivo bone bioactivity?. Biomaterials.

[16] Li X., Wang X., Zhao T., Gao B., Miao Y., Zhang D., Dong Y. (2014). Guided bone regeneration using chitosan-collagen membranes in dog dehiscence-type defect model. J. Oral Maxillofac. Surg..

[17] Coleman N.J., Bellantone M., Nicholson J.W., Mendham A.P. (2007). Textural and structural properties of bioactive glasses in the system CaO-SiO_2_. Ceramics Silikáty.

[18] Lee B.-S., Lin H.-P., Chan J.C.-C., Wang W.-C., Hung P.-H., Tsai Y.-H., Lee Y.-L. (2018). A novel sol-gel-derived calcium silicate cement with short setting time for application in endodontic repair of perforations. Int. J. Nanomed..

[19] Yu B., Turdean-Ionescu C.A., Martin R.A., Newport R.J., Hanna J.V., Smith M.E., Jones J.R. (2012). Effect of calcium source on structure and properties of sol-gel derived bioactive glasses. Langmuir.

[20] Chang Y.-Y., Huang H.-L., Chen Y.-C., Hsu J.-T., Shieh T.-M., Ming-Tzu Tsai M.-T. (2014). Biological Characteristics of the MG-63 human osteosarcoma cells on composite tantalum carbide/amorphous carbon films. PLoS ONE.

[21] Turco G., Porrelli D., Marsich E., Vecchies F., Lombardi T., Stacchi C., Di Lenarda R. (2018). Three-dimensional bone substitutes for oral and maxillofacial surgery: Biological and structural characterization. J. Funct. Biomater..

[22] Catauro M., Bollino F., Papale F. (2014). Preparation, characterization, and biological properties of organic-inorganic nanocomposite coatings on titanium substrates prepared by sol-gel. J. Biomed. Mater. Res. Part A.

[23] Qasim S.B., Delaine-Smith R.M., Fey T., Rawlinson A., Rehman I.U. (2015). Freeze gelated porous membranes for periodontal tissue regeneration. Acta Biomater..

[24] Hurt A.P., Kotha A.K., Trivedi V., Coleman N.J. (2015). Bioactivity, biocompatibility and antimicrobial properties of a chitosan-mineral composite for periodontal tissue regeneration. Polimeros.

[25] Staehlke S., Rebl H., Nebe B. (2019). Phenotypic stability of the human MG-63 osteoblastic cell line at different passages. Cell Biol. Int..

[26] Brinker J., Scherer G.W. (1990). Sol-Gel Science. The Physics and Chemistry of Sol-Gel Processing.

[27] Li P.J., Ohtsuki C., Kokubo T., Nakanishi K., Soga N., de Groot K. (1994). The role of hydrated silica, titania, and alumina in inducing apatite on implants. J. Biomed. Mater. Res..

[28] Lutz-Christian Gerhardt L.-C., Boccaccini A.R. (2010). Bioactive glass and glass-ceramic scaffolds for bone tissue engineering. Materials.

[29] Fiume E., Barberi J., Verné E., Baino F. (2018). Bioactive glasses: From parent 45S5 composition to scaffold-assisted tissue-healing therapies. J. Funct. Biomater..

[30] Diab R., Canilho N., Pavel I.A., Haffner F.B., Girardon M., Pasc A. (2017). Silica-based systems for oral delivery of drugs, macromolecules and cells. Adv. Colloid Interface Sci..

[31] Labbaf S., Tsigkou O., Mueller K.H., Stevens M.M., Porter A.E., Jones J.R. (2011). Spherical bioactive glass particles and their interaction with human mesenchymal stem cells in vitro. Biomaterials.

[32] Lin S., Ionescu C., Pike K.J., Smith M.E., Jones J.R. (2009). Nanostructure evolution and calcium distribution in sol-gel derived bioactive glass. J. Mater. Chem..

[33] Valliant E.M., Turdean-Ionescu C.A., Hanna J.V., Smith M.E., Jones J.R. (2012). Role of pH and temperature on silica network formation and calcium incorporation into sol–gel derived bioactive glasses. J. Mater. Chem..

[34] Luz G.M., Mano J.F. (2011). Preparation and characterization of bioactive glass nanoparticles prepared by sol–gel for biomedical applications. Nanotechnology.

[35] Xia W., Chang J. (2007). Preparation and characterization of nano-bioactive-glasses (NBG) by a quick alkali-mediated sol-gel method. Mater. Lett..

[36] Itala A., Ylanen H.O., Yrjans J., Heino T., Hentunen T., Hupa M., Aro H.T. (2002). Characterization of microrough bioactive glass surface: Surface reactions and osteoblast responses in vitro. J. Biomed. Mater. Res..

[37] Gough J.E., Notingher I., Hench L.L. (2004). Osteoblast attachment and mineralized nodule formation on rough and smooth 45S5 bioactive glass monoliths. J. Biomed. Mater. Res..

[38] Tolb E., Müller W.E.G., Abd El-Hady B.M., Neufurth M., Wurm F., Wang S., Schröder H.C., Wang X. (2016). High biocompatibility and improved osteogenic potential of amorphous calcium carbonate/vaterite. J. Mater. Chem. B.

[39] Lide D.R. (2006). CRC Handbook of Chemistry and Physics.

[40] Lee E.-J., Shin D.-S., Kim H.-E., Kim H.-W., Koh Y.-H., Jang J.-H. (2009). Membrane of hybrid chitosan-silica xerogel for guided bone regeneration. Biomaterials.

[41] Zhang Y., Zhang M. (2001). Synthesis and characterization of macroporous chitosan/calcium phosphate composite scaffolds for tissue engineering. J. Biomed. Mater. Res..

[42] Pattnaik S., Nethala S., Tripathi A., Saravanan S., Moorthi A., Selvamurugan N. (2011). Chitosan scaffolds containing silicon dioxide and zirconia nanoparticles for bone tissue engineering. Int. J. Biol. Macromol..

[43] Rodrigues J.R., Alves N.M., Mano J.F. (2016). Biomimetic polysaccharide/bioactive glass nanoparticles multilayer membranes for guided tissue regeneration. RSC Adv..

[44] Wang Y.Y., Papagerakis S., Faulk D., Badylak S.F., Zhao Y.M., Ge L.H., Qin M., Papagerakis P. (2018). Extracellular matrix membrane induces cementoblastic/osteogenic properties of human periodontal ligament stem cells. Front. Physiol..

[45] Narayanan G., Bhattacharjee M., Nair L.S., Laurencin C.T. (2017). Musculoskeletal tissue regeneration: The role of the stem cells. Regen. Eng. Transl. Med..

[46] Carvalho S.M., Oliveira A.A.R., Jardim C.A., Melo C.B.S., Gomes D.A., de Fátima Leite M., Pereira MM. (2012). Characterization and induction of cementoblast cell proliferation by bioactive glass nanoparticles. J. Tissue Eng. Regen. Med..

[47] Perkin F.M., Pratt L. (1909). Action of alcohols on metallic calcium. J. Chem. Soc. Trans..

